# Triglyceride-glucose (TyG) index is a predictor of arterial stiffness, incidence of diabetes, cardiovascular disease, and all-cause and cardiovascular mortality: A longitudinal two-cohort analysis

**DOI:** 10.3389/fcvm.2022.1035105

**Published:** 2023-01-04

**Authors:** Iram Faqir Muhammad, Xue Bao, Peter M. Nilsson, Suneela Zaigham

**Affiliations:** ^1^Department of Clinical Sciences, Lund University, Malmö, Sweden; ^2^Department of Cardiology, Nanjing Drum Tower Hospital, The Affiliated Hospital of Nanjing University Medical School, Nanjing, China; ^3^Department of Internal Medicine, Skåne University Hospital, Malmö, Sweden; ^4^Department of Medical Sciences, Uppsala University, Uppsala, Sweden

**Keywords:** arterial stiffness, cardiovascular disease, cardiovascular mortality, diabetes, insulin resistance, mortality, triglyceride-glucose index

## Abstract

**Background:**

Triglyceride-glucose (TyG) index is a useful low-cost marker of insulin resistance. We aimed to evaluate the association between TyG index and arterial stiffness, incidence of diabetes, adverse cardiovascular outcomes, and all-cause and cardiovascular mortality in two large prospective Swedish cohorts, the Malmö Diet and Cancer Study-Cardiovascular Cohort (MDCS-CV) and the Malmö Preventive Project (MPP).

**Methods:**

Association between baseline TyG index and arterial stiffness, measured by carotid femoral pulse wave velocity (c-f PWV), was assessed using linear regression and general linear models, adjusting for covariates. Cox proportional hazard regression was used to assess the association between TyG index and incidence of diabetes, coronary events (CE), stroke, atrial fibrillation (AF), heart failure, and all-cause and cardiovascular mortality.

**Results:**

After multivariable adjustment, baseline TyG index was significantly associated with increased arterial stiffness (β for c-f PWV = 0.61, *p* = 0.018). Participants in the highest quartile of TyG index vs. lowest quartile had an increased incidence of diabetes (HR: 3.30, 95% CI: 2.47–4.41), CE (HR: 1.53, 95% CI: 1.41–1.68), stroke (HR: 1.30, 95% CI: 1.18–1.44), all-cause mortality (HR: 1.22, 95% CI: 1.16–1.28), and cardiovascular mortality (HR: 1.37, 95% CI: 1.26–1.49) after adjustment for covariates. Per unit increase in TyG index was associated with increased heart failure risk. No significant association was observed for incident AF.

**Conclusion:**

Elevated TyG index is positively associated with increased arterial stiffness and increased incidence of diabetes, CE, stroke, and all-cause and cardiovascular mortality. The results suggest that TyG index can potentially be useful in the identification of those at increased long-term risk of adverse health outcomes.

## Introduction

Hyperglycemia is known to be a main driver for the development of diabetes and cardiovascular diseases (CVD). Insulin is closely related to glucose and lipid metabolism. Insulin resistance (IR), reflecting the insensitivity state of the peripheral tissue toward insulin, leads to defective glucose uptake, decreased glycogenesis and dyslipidemia (1). IR has been shown to have a close relationship with adverse cardiovascular and metabolic outcomes (1, 2). Thus, identifying those with IR could be of value for early risk stratification and clinical management.

Triglyceride-glucose index (TyG) is a surrogate marker for IR and is readily calculated using fasting measurements of plasma triglyceride and glucose, which are routine clinical lab investigations. TyG index has shown to be correlated with euglycemic-hyperinsulinemic clamp test, which is considered the gold standard for determining insulin sensitivity (3), but it is a costly and invasive method requiring trained staff. Another frequently used approach to determine IR is using the homeostasis model assessment of insulin resistance (HOMA-IR), which requires insulin levels for calculation. However, as insulin levels are not routinely measured in the clinical setting, the wider application of this measure outside research settings is limited. TyG index has been shown to correlate with HOMA-IR (4) and is therefore a useful surrogate tool to identify those with IR.

Triglyceride-glucose index has shown to have a close association with a range of adverse health risk factors such as obesity and hypertriglyceridemia (5–7), indicating its potential impact on cardiometabolic health. Moreover, the role of TyG index in CVD has been explored in those with diabetes but not widely so in the general population. We aimed to investigate whether TyG index is associated with increased risk of diabetes, arterial stiffness, and CVD including coronary events (CE), stroke, atrial fibrillation (AF), heart failure, and all-cause as well as cardiovascular mortality in the general population.

## Materials and methods

### Study population

Data for this analysis was used from the Malmö Diet and Cancer Study-Cardiovascular Cohort (MDCS-CV) and the Malmö Preventive Project (MPP), two prospective population-based cohort studies from an urban area in the south of Sweden.

The MDCS study is a large prospective cohort, comprising of men and women from the city of Malmö, Sweden (8). From this cohort, a random sample of participants were re-invited between November 1991 and February 1994 to investigate the epidemiology of carotid artery abnormalities. This sub-cohort was called the MDCS-CV and comprised of 6,103 participants (2,572 men and 3,531 women) (9). Between May 2007 and January 2012, a re-examination of participants from the sub-cohort was carried out. Follow-up data from 3,734 participants (76% participation rate) who attended the re-examination was attained (10). Measurements of carotid femoral pulse wave velocity (c-f PWV) was carried out during the re-examination and data was available for 3,056 participants (10). Participants with missing data on triglycerides, glucose and key covariates (*n* = 359) were excluded, resulting in a final study population of 2,697 subjects.

For analysis for incident diabetes, we excluded participants with, use of antidiabetic medication, self-reported diabetes, or with fasting blood glucose ≥6.1 mmol/L [corresponding to a fasting plasma glucose cut-off of 7.0 mmol/L indicating a diagnosis of diabetes (*n* = 558)]. Participants with missing values of triglycerides and glucose (*n* = 569) and for other covariates (*n* = 405) were also excluded. The final study population was 4,571.

The study population for the analyses in MDCS-CV is shown in Supplementary Figure 1.

The MPP consisted of 22,444 men and 10,902 women (33,346 participants in total) who were recruited by pre-specified birth year groups from Malmö city to take part in a health examination. Participants with missing data on triglycerides and glucose (*n* = 225) and other key covariates (*n* = 161) were excluded. We also excluded cases of prevalent cardiovascular disease, i.e., CE, stroke, AF, and heart failure, for each of their respective analysis. The study population flow chart for the analyses in MPP is illustrated in Supplementary Figure 2.

The Regional Ethics Review Board in Lund approved the study (LU 51-90, LU 532-2006, LU 85-2004, and LU 2011-412) and the participants provided written informed consent in MDCS-CV and verbal informed consent in MPP. The study was conducted in accordance with the Helsinki Declaration.

### Baseline examinations

#### MDCS-CV

The baseline examinations comprised of physical examination, blood sample collection, and a self-administered questionnaire. Information about smoking habits, leisure-time physical activity, and use of antihypertensive medication was collected from the questionnaire. A total physical activity score was calculated by multiplying the duration of specific activities by the corresponding intensity coefficient. Smoking status was categorized into three groups: former smokers, non-smokers, and current smokers. Blood pressure (mmHg) was measured once, after 10 min of rest, while the subject was in supine position using a mercury-column sphygmomanometer. Waist circumference (cm) was determined midway between the lowest rib margin and the iliac crest. Height (cm) was measured by using a fixed stadiometer. A calibrated balance-beam scale was used to measure weight (kg), with the participants wearing light clothing and no shoes. BMI was calculated as kg/m^2^. Blood glucose (mmol/L), triglycerides (mmol/L) and high-density lipoprotein cholesterol (HDL-C; mmol/L) were determined from fasting blood samples, using standardized procedures at the Department of Clinical Chemistry, Skåne University Hospital. Low-density lipoprotein cholesterol (LDL-C; mmol/L) was calculated using Friedewald’s formula. Insulin (mIU/L) was analyzed by using radioimmunoassay. C-reactive protein (CRP; mg/L) was determined with a Tina-quant CRP latex assay (Roche Diagnostics, Basel, Switzerland) (10). HOMA2-IR was calculated with the use of a HOMA2-IR calculator (11).

#### MPP

Blood samples were taken after an overnight fast and were analyzed using standard procedures at the Department of Clinical Chemistry, Malmö University Hospital to determine triglycerides, cholesterol, and glucose. Fasting blood glucose was analyzed using two methods during the different study times: the glucose-oxidase method (1974–1977) or the hexokinase-oxidase method (1977–1992). As the two methods provide similar results, no conversion factor was used. Height (m) was measured standing without shoes using a fixed stadiometer. Weight (kg) was measured on a balance beam scale with light indoor clothing. BMI was calculated as kg/m^2^. Blood pressure (mmHg) was measured twice after 10 min rest in the supine position using a sphygmomanometer. Information about smoking status, anti-hypertensive medication, alcohol intake and physical activity was gathered from a self-administered questionnaire. Smoking status was categorized into two categories; current smokers, and non-smokers and ex-smokers. Prevalent diabetes information was retrieved using self-reported diabetes at baseline, prior diagnosis of diabetes in hospital or other registers, or fasting whole blood glucose ≥6.1 mmol/L at baseline (corresponding to a plasma glucose of ≥7.0 mmol/L) (10).

#### TyG index

For analysis in both cohorts, TyG index was calculated using the following formula (5, 12): Ln(fasting triglycerides (mg per dl) × fasting glucose (mg per dl))/2.

### Endpoint ascertainment

#### MDCS-CV

##### Arterial stiffness measurement

Arterial stiffness was measured in 2007–2012 as c-f PWV using an applanation tonometry technique (SphygmoCor, Atcor Medical, Australia) and has been described in detail previously (13). The participants were asked to rest in a supine position for 5 min, after which pulse curves from the carotid and femoral arteries were obtained using a pressure-sensitive probe. The distance was measured from the suprasternal notch to the umbilicus and from the umbilicus to the measuring point at the femoral artery, subtracting the distance between the suprasternal notch and the measuring point at the carotid artery. The time from the peak of the R-wave on the electrocardiogram to the foot of the pulse wave at the carotid and femoral arteries was automatically calculated by using the simultaneously registered electrocardiogram (13). Every participant had a varying number of successful measurements (ranging between one and five). The aim was to achieve three measurements in each (possible in 86.7% of the case subjects). Mean c-f PWV was calculated from these measurements. The formula (2 × diastolic pressure + systolic pressure)/3 was used to calculate mean arterial pressure (MAP) (13).

##### Incidence of diabetes

All participants free of diabetes (*n* = 4,571) were followed from the baseline measurements until first incidence of diabetes, emigration from Sweden, death, or the end of follow-up (31 December 2020), whichever came first. Both local and national registers were used to identify incident diabetes cases, and have been explained in detail previously (14). Briefly, incident diabetes information was collected from six sources: the Swedish National Diabetes Register, the regional Diabetes 2000 register of the Scania region, the Swedish Inpatient Register, the Swedish Outpatient Register, the Malmö HbA1c register and the nationwide Swedish Drug Prescription Register. New cases of diabetes were diagnosed according to established criteria (fasting plasma glucose concentration ≥7.0 mmol/L resulting from two repeated tests on separate occasions) in the Swedish National Diabetes Register and the Diabetes 2000 register. In the Malmö local HbA1c register, subjects were diagnosed with diabetes if they had at least two HbA1c recordings ≥42 mmol/mol (6.0%), based on the Swedish Mono-S-based standardization system [corresponding to 53 mmol/mol (7.0%), according to the U.S. National Glycohemoglobin Standardization Program]. In the Swedish inpatient and outpatient registers, a senior physician diagnosed diabetes. A filled prescription of insulin or glucose lowering medication (Anatomical Therapeutic Chemical Classification System code A10) was required for a diagnosis of diabetes in the nationwide prescription register (13).

#### MPP

##### Incidence of cardiovascular disease and mortality

Participants in MPP were followed from baseline examinations until diagnosis of studied outcome, emigration from Sweden or end of follow-up (31 December 2019), whichever came first. For analysis of each of the cardiovascular outcomes, the respective prevalent cases were excluded. Endpoints were ascertained by data linkages to local and national registers. The studied outcomes were incident CE (ICD-9 codes: 410–414), incident stroke (ICD-9 codes: 430, 431, 434, 436 and 23 unknown cases), incident AF (ICD-9 codes: 427D), and incident heart failure (ICD-9 codes: 428, ICD-10 codes: 428 and I11.0). Information about all-cause and cardiovascular mortality was retrieved from the national Swedish cause of death register. Cardiovascular mortality as underlying cause of death was defined as ICD-9 codes: 390–459 or ICD-10 codes: C00-D48.

### Statistical analysis

C-reactive protein and HOMA-IR in the MDCS-CV were naturally log-transformed due to their skewed distribution. Descriptive data for the study population was reported as means ± SD, median (25th–75th percentiles) or proportions (percentage), as appropriate. The differences across the baseline characteristics were tested using χ^2^ for categorical variables and Analysis of Variance (ANOVA) for continuous variables.

Quartiles of TyG index were created with the lowest quartile (Q1) as the reference category. Linear regression and univariate general linear models were used to explore the association between TyG index and c-f PWV as the dependent variable. Cox proportional hazard regression was used to estimate the hazard ratios (HRs) with corresponding 95% confidence intervals (CIs) to assess the association across the quartiles of TyG index and incidence of diabetes, CE, stroke, AF, heart failure, and all-cause and cardiovascular mortality. Time to follow-up was used as time scale. Proportional hazard assumptions were tested by incorporating the time-dependent effects of covariates. Some deviation from assumption was observed in case of incident CE, stroke and heart failure. The analyses were, therefore, conducted again for follow-up time intervals before and after the median. However, this showed that the effect of this deviation on HRs was minor. Adjustments were done for potential confounders, which were selected based on factors in the literature that are known to affect the association between TyG index and the various health outcomes. For incident diabetes, three incremental models were fitted: *Model 1* was adjusted for age and sex. *Model 2* was further adjusted for waist, systolic blood pressure, HDL-C, LDL-C, smoking status, anti-hypertensive medication, and lipid-lowering medication. Finally, *Model 3* was additionally adjusted for CRP. In the analysis for c-f PWV, additional adjustments for age at the time of measurement of c-f PWV, MAP, heart rate, and prevalent diabetes were carried out.

For the analysis in MPP, the multivariable analyses included adjustments for age, sex, BMI, smoking status, total cholesterol, diabetes, anti-hypertensive medication, physical activity, and alcohol habits in a series of models. Kaplan–Meier curves were used to plot the incidence of all health outcomes across the quartiles of TyG index.

We also conducted several sensitivity analysis. Information for insulin and HOMA-IR was available for the MDCS-CV cohort. In a sensitivity analysis, we repeated the analyses using HOMA-IR in place of TyG index as a surrogate marker for IR to test the association with arterial stiffness. As obesity is closely related to diabetes and IR, we explored the associations stratified for BMI to test the predictive ability of TyG index in non-obese participants as well. BMI cut-off values recommended by WHO were used to categorize participants into three categories, i.e., normal weight (BMI <25), overweight (BMI ≥25 to <30), and obese (BMI ≥30 kg/m^2^) (15). We also examined the association by testing for interaction for age, sex, and BMI. Furthermore, in an exploratory analysis, the study population was stratified for age, sex and BMI for all health outcomes. We also calculated area under the curve (AUC) for TyG index in relation to CVD mortality, all-cause mortality and incident diabetes.

All analyses were performed using SPSS version 27 (IBM Corp., Armonk, NY, USA). A two-tailed *p*-value of <0.05 was regarded as statistically significant.

## Results

### Baseline characteristics

The baseline characteristics of the participants in the MDCS-CV and MPP are presented in Supplementary Tables 1, 2, respectively. The mean age of the MDCS-CV and the MPP cohorts at baseline were 58 and 46 years, respectively. In MPP, participants with higher TyG index were more likely to be men, had higher systolic blood pressure, less physically active, more likely to be smokers, and had a higher BMI compared with other participants.

### Arterial stiffness

The mean follow-up time from baseline examinations to the c-f PWV measurement was 16.9 ± 1.5 years. There was a significant association between baseline TyG index and c-f PWV (β = 0.61, *p* = 0.018), after adjustments in the final *Model 3* (Supplementary Table 3). When the quartiles of TyG index were compared, c-f PWV was significantly higher for the participants in the fourth quartile vs. first quartile (11.0 vs. 10.93 m/s) (*p* < 0.001) in *Model 1*. The association remained significant after adjustment for potential confounders as shown in Table 1. In an additional sensitivity model, when further adjustments were done for physical activity, the results remained essentially unchanged (results not shown). When the analyses was conducted using HOMA-IR in place of TyG index, no significant association was observed (results not shown).

**TABLE 1:** Association between quartiles of TyG-index and c-f PWV (*n* = 2,697).

TyG index	Q1	Q2	Q3	Q4	*p* for trend
Participants, *n*	675	672	675	675	
Model 1	10.93	10.43**	10.45**	11.00***	<0.001
Model 2	10.27	10.53*	10.45	10.74***	0.001
Model 3	10.33	10.55*	10.43	10.67*	0.039
Model 4	10.33	10.55*	10.42	10.68*	0.030

Model 1: Adjusted for sex, MAP, heart rate and age at follow-up, and age at baseline. Model 2: Adjusted for sex, MAP, heart rate and age at follow-up, baseline age, smoking habits, systolic blood pressure, waist circumference, diabetes, use of anti-hypertensive medication, and use of lipid-lowering medication. Model 3: Adjusted for sex, MAP, heart rate and age at follow-up, baseline age, smoking habits, systolic blood pressure, waist circumference, diabetes, use of anti-hypertensive medication, use of lipid-lowering medication, HDL, and LDL. Model 4: Model 3 + CRP.

*p < 0.05, **p < 0.01, ***p < 0.001.

### Incidence of diabetes

**FIGURE 1:**
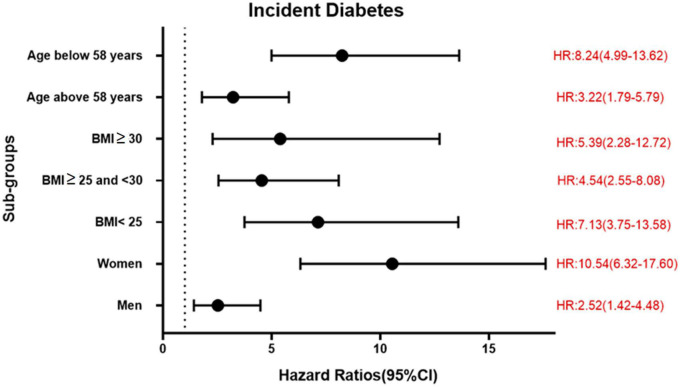
Association between per unit increase of TyG index and incident diabetes in different sub-groups in MDCS-CV.

**FIGURE 2:**
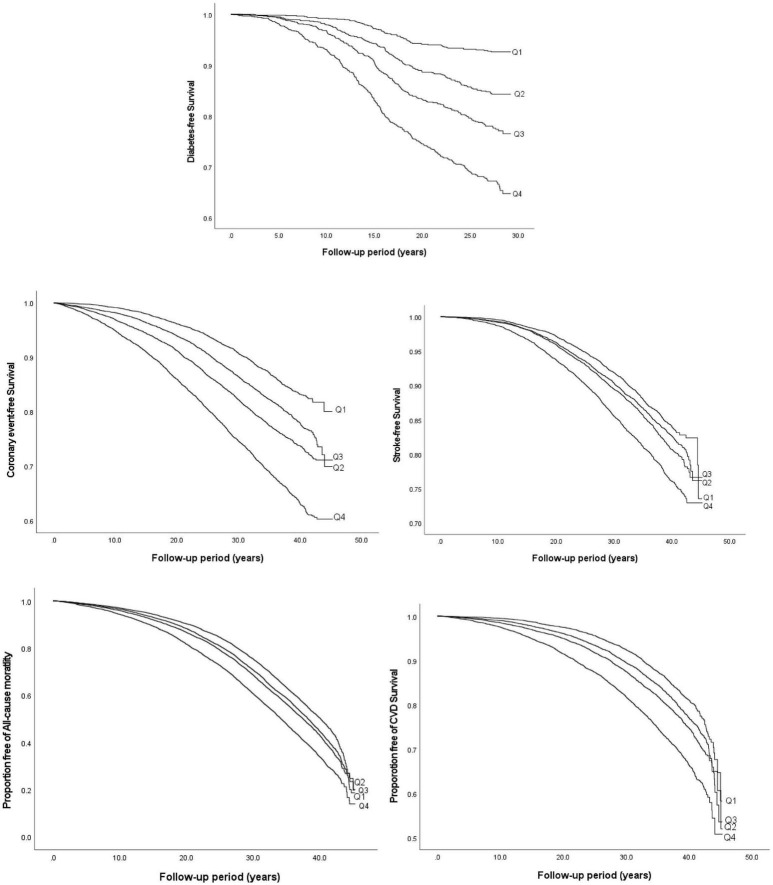
Kaplan–Meier curves of incident diabetes, CVD, all-cause, and cardiovascular mortality by quartiles of TyG index.

During a mean follow-up time of 21.2 ± 7.4 years in the MDCS-CV, there were 754 cases of incident diabetes. HRs for incident diabetes are presented in Table 2. After all the adjustments in *Model 3*, higher TyG index was associated with a significantly higher risk of diabetes incidence (HR: 3.30, 95% CI: 2.47–4.41) (*p* for trend <0.001). The HR for incident diabetes per unit increase in TyG index was 5.53 (95% CI: 3.78–8.09) in the Model 3. The results remained changed when further adjustments were done for physical activity (results not shown). There was no significant interaction with age and BMI but significant interactions with sex were observed. In the analysis stratified by BMI, increasing TyG index quartiles were significantly associated with greater risk of incidence of diabetes across all BMI categories. Overall, HR for incident diabetes for participants per unit increase in TyG index was 7.13 (95% CI: 3.75–13.58) for normal weight, 4.54 (95% CI: 2.55–8.08) for over-weight, and 5.39 (95% CI: 2.28–12.72) for obese participants. The age, sex, and BMI stratified HR are presented in Figure 1. The Kaplan–Meier estimates for incident diabetes were plotted in Figure 2.

**TABLE 2:** Incidence of diabetes in relation to quartiles of TyG index in MDC-CC (*n* = 4,571, incident diabetes, *n* = 754).

TyG index	Q1	Q2	Q3	Q4	*p* for trend	Per unit
Participants, *n*	1,147	1,140	1,140	1,144		
Incidence of diabetes, *n* (per 1,000 person-years)	75 (2.67)	149 (5.75)	215 (8.68)	315 (13.84)		
Model 1	1	2.19 (1.66–2.90)***	3.36 (2.58–4.38)***	5.47 (4.23–7.08)***	<0.001	12.18 (8.97–16.55)***
Model 2	1	1.85 (1.39–2.45)***	2.44 (1.84–3.22)***	3.27 (2.44–4.37)***	<0.001	5.36 (3.66–7.84)***
Model 3	1	1.86 (1.40–2.46)***	2.43 (1.84–3.21)***	3.30 (2.47–4.41)***	<0.001	5.53 (3.78–8.09)***

Model 1: Age and sex. Model 2: Age, sex smoking status, waist, antihypertensive medication, lipid-lowering medication, HDL, LDL, and systolic blood pressure. Model 3: Model 2 + CRP.

*p < 0.05, **p < 0.01, ***p < 0.001.

### Incidence of CVD

**FIGURE 3:**
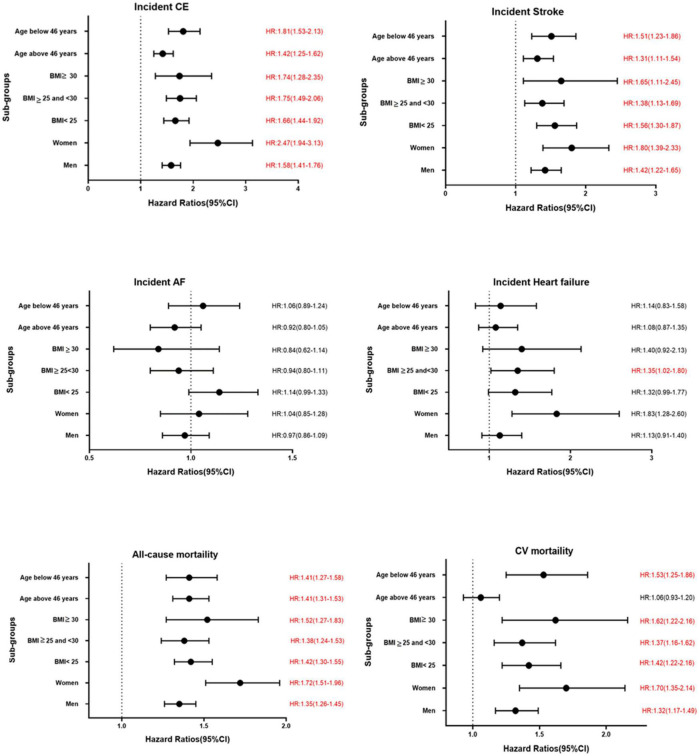
Association between per unit increase of TyG index and various CVD outcomes in different sub-groups in MPP. Significant results are presented in red.

The results of the Cox proportional hazard regression to assess the association between TyG index and various cardiovascular outcomes are reported in Table 3. Significant association was observed between TyG index and incident CE and stroke in the highest quartile and per unit increase in TyG index, and with heart failure for per unit increase in TyG index after adjusting for potential confounders. No association was observed with AF. Compared to the reference category (Q1), HRs (95% CI) for incident CE and stroke for individuals in the fourth quartile of TyG index were 1.53 (1.41–1.68) and 1.30 (1.18–1.44), respectively, after adjustments in the final *Model 3* (Table 3). A significant interaction was observed between sex and TyG index for incident CE. No significant interaction was observed between age, sex, BMI, and TyG index in relation to stroke or heart failure. Kaplan–Meier curves for incident CVD outcomes are shown in Figure 2. Stratified results for the CVD outcomes are presented in Figure 3.

**TABLE 3:** Incidence of CVD events and all-cause as well as CV mortality in relation to quartiles of TyG index in MPP.

TyG index	Q1	Q2	Q3	Q4	*p* for trend	Per unit
**Cardiac events (6,402/32,838)**						
Participants, *n*	8,224	8,198	8,205	8,211		
Incidence of CE, *n* (per 1,000 person-years)	988 (3.80)	1,379 (5.43)	1,717 (6.90)	2,318 (10.07)		
Model 1	1	1.34 (1.24–1.46)***	1.68 (1.55–1.82)***	2.40 (2.22–2.59)***	<0.001	3.25 (2.99–3.54)***
Model 2	1	1.18 (1.09–1.28)***	1.34 (1.24–1.46)***	1.55 (1.42–1.68)***	<0.001	1.73 (1.56–1.91)***
Model 3	1	1.17 (1.08–1.27)***	1.33 (1.23–1.44)***	1.53 (1.41–1.68)***	<0.001	1.72 (1.55–1.90)***
**Stroke (4,385/32,920)**						
Participants, *n*	8,228	8,240	8,211	8,241		
Incidence of stroke, *n* (per 1,000 person-years)	909 (3.50)	1,026 (4.01)	1,114 (4.41)	1,336 (5.61)		
Model 1	1	1.14 (1.04–1.25)***	1.27 (1.16–1.39)***	1.65 (1.52–1.80)***	<0.001	2.12 (1.90–2.36)***
Model 2	1	1.07 (0.98–1.17)	1.13 (1.03–1.24)**	1.31 (1.19–1.44)***	<0.001	1.53 (1.35–1.74)***
Model 3	1	1.07 (0.98–1.17)	1.13 (1.03–1.24)**	1.30 (1.18–1.44)***	<0.001	1.52 (1.33–1.72)***
**Atrial fibrillation (6,950/32,917)**						
Participants, *n*	8,221	8,232	8,232	8,214		
Incidence of AF, *n* (per 1,000 person-years)	1,613 (6.34)	1,766 (7.02)	1,753 (7.01)	1,813 (7.68)		
Model 1	1	1.10 (1.03–1.18)**	1.11 (1.04–1.19)**	1.26 (1.18–1.35)***	<0.001	1.45 (1.32–1.58)***
Model 2	1	1.01 (0.94–1.08)	0.95 (0.89–1.02)	0.95 (0.88–1.02)	0.063	0.97 (0.88–1.08)
Model 3	1	1.02 (0.95–1.09)	0.97 (0.90–1.04)	0.96 (0.89–1.04)	0.142	0.99 (0.89–1.11)
**Heart failure (2,105/32,831)**						
Participants, *n*	8,223	8,197	8,203	8,208		
Incidence of heart failure, *n* (per 1,000 person-years)	425 (1.65)	503 (2.00)	521 (2.11)	656 (2.88)		
Model 1	1	1.22 (1.16–1.51)**	1.32 (1.16–1.51)***	1.92 (1.69–2.17)***	<0.001	2.74 (2.35–3.20)***
Model 2	1	1.04 (0.92–1.19)	1.01 (0.88–1.16)	1.14 (0.99–1.31)	0.112	1.33 (1.11–1.60)**
Model 3	1	1.04 (0.91–1.18)	1.00 (0.88–1.15)	1.12 (0.97–1.29)	0.166	1.30 (1.08–1.56)**
**All-cause mortality (17,916/32,960)**						
Participants, *n*	8,236	8,248	8,250	8,226		
Incidence of all-cause mortality, *n* (per 1,000 person-years)	3,612 (13.59)	4,321 (16.37)	4,629 (17.62)	5,354 (21.61)		
Model 1	1	1.18 (1.13–1.24)***	1.28 (1.22–1.34)***	1.61 (1.54–1.68)***	<0.001	2.07 (1.96–2.19***
Model 2	1	1.09 (1.04–1.14)***	1.12 (1.07–1.18)***	1.25 (1.19–1.31)***	<0.001	1.47 (1.38–1.57)***
Model 3	1	1.08 (1.04–1.13)***	1.12 (1.07–1.17)***	1.22 (1.16–1.28)***	<0.001	1.42 (1.34–1.52)***
**Cardiovascular (CV) mortality (6,160/32,960)**						
Participants, *n*	8,236	8,248	8,250	8,226		
Incidence of CV mortality, *n* (per 1,000 person-years)	1,077 (4.05)	1,390 (5.27)	1,610 (6.13)	2,083 (8.40)		
Model 1	1	1.25 (1.15–1.35)***	1.44 (1.33–1.56)***	2.00 (1.86–2.16)***	<0.001	2.85 (2.61–3.11)***
Model 2	1	1.12 (1.03–1.21)***	1.21 (1.12–1.31)***	1.40 (1.29–1.52)***	<0.001	1.74 (1.57–1.92)***
Model 3	1	1.11 (1.02–1.20)***	1.19 (1.10–1.29)***	1.37 (1.26–1.49)***	<0.001	1.68 (1.52–1.86)***

Model 1: Age and sex. Model 2: Age, sex, BMI, systolic blood pressure, cholesterol, smoking status, diabetes, and antihypertensive medication. Model 3: Model 2 + physical activity and alcohol.

*p < 0.05, **p < 0.01, ***p < 0.001.

### All-cause and cardiovascular mortality

Triglyceride-glucose index was significantly associated with all-cause and cardiovascular mortality. After multivariable adjustment, the HR for all-cause mortality for the participants in the fourth quartile (Q4) of TyG index vs. Q1 was 1.22 (95% CI: 1.16–1.28) (Table 3). Per unit increase in TyG index, the HR was 1.42 (1.34–1.52) for the final multivariable adjusted model. For cardiovascular mortality, the risk was significantly increased with higher TyG index (HR for Q4 vs. Q1: 1.37, 95% CI: 1.26–1.49). Significant interactions were observed for sex and BMI, but not for age for all-cause mortality, and only with BMI for cardiovascular mortality. When stratified for BMI categories, mean age and gender, the results remained significant for both men and women and across all categories of BMI and age after adjustments. Similar results were observed for cardiovascular mortality (Figure 3). Figure 2 shows Kaplan–Meier curves for all-cause and cardiovascular mortality.

The AUC of TyG index for CVD mortality, all-cause mortality, and incident diabetes was 0.59, 0.59, and 0.71, respectively (all *p* < 0.001).

## Discussion

In this large population-based study based on two cohorts, TyG index was an independent predictor for increased arterial stiffness, incident diabetes, CE, stroke, and heart failure. We also found that TyG index was associated with increased risk of all-cause and cardiovascular mortality in the general population. There was, however, no significant association with incident AF.

Although diabetes is a major risk factor for CVD, disturbances in metabolism in advance of diabetes may have a role to promote CVD. Triglycerides have been shown to be associated with arterial stiffness (15). Arterial stiffness has also shown to be elevated in individuals with metabolic syndrome (16). Few studies have looked at the association between TyG index and arterial stiffness, when for example brachial-ankle PWV (baPWV) has been used as a measure of arterial stiffness (17). Yan et al. assessed the longitudinal relationship between TyG index and baPWV but were limited by the size of the study population (18). The results of our study show that IR may lead to higher c-f PWV in the general population. In a cross-sectional study, Wang et al. (19) found an association between TyG index and baPWV in patients with type 2 diabetes, but not with HOMA-IR. Similarly, we found TyG index to be associated with arterial stiffness after follow-up whereas no significant association was observed between HOMA-IR and arterial stiffness. Although vascular aging is a natural phenomenon, the process is accelerated with increasing age, obesity and in the presence of diabetes as well as IR. One mechanism linked with increased arterial stiffness in an IR state is endothelial cell dysfunction and vascular smooth muscle cell stiffness (20), of which hyperglycemia is a key driving factor. As arterial stiffness is an established determinant of cardiovascular events and all-cause mortality (21), association of TyG index would be of interest to improve risk stratification. In our study, the difference in c-f PWV between the fourth quartile vs. first quartile of TyG index, though statistically significant, was marginal. However, it shows how the high risk in those with high TyG index develops over time, and hence may be of clinical relevance.

Insulin resistance is a key pathological feature of type 2 diabetes. It is closely related to obesity and precedes the development of diabetes (22). The results of our study show that TyG index was associated with a higher risk of incident diabetes, even among normal weight individuals. Hence, the TyG index can be a useful predictor for incident diabetes even in non-obese individuals. This confirms the findings of a recent meta-analysis that demonstrated an association of TyG index with risk of future diabetes (23). Pancreatic β-cell dysfunction is another pathological feature of type 2 diabetes. There also exists a complex relation between IR and β-cell dysfunction (24). In brief, IR and β-cell dysfunction are pivotal characteristics of future type 2 diabetes. Thus, TyG index, a surrogate marker for IR, could be of considerable clinical use to identify those at risk of developing type 2 diabetes. Moreover, obesity is a major driving feature of IR, but stratifying the study population by BMI showed that TyG index retained predictive value across all strata, and therefore, is independent of obesity.

Epidemiological data has shown that TyG index is associated with CVD in high-risk populations. Previously it was observed that IR is associated with adverse cardiovascular events in patients with coronary artery disease (25), hypertensive patients (26), or non-ST elevation acute coronary syndrome (27). Our results are in line with previous studies, which demonstrated association of TyG index with adverse cardiovascular events (11, 28). A recent meta-analysis reported an independent association between higher TyG index with an increased risk of incidence of atherosclerotic CVD (29). Increased risk for heart failure was found for per unit increase in TyG index. Though association between IR and heart failure is well accepted (30), results in the general population are limited. One study explored relationship between TyG index as a marker for IR and heart failure, but these authors did not observe a significant association (31). However, a recent study from the Atherosclerosis Risk in Communities study (ARIC) showed a significant association between TyG index and incident HF (32).

We did not find any association of TyG index with AF in our study population. Previous studies looking at this association have been carried out in high-risk patient groups. One study showed an association of TyG index with AF in patients undergoing septal myectomy for hypertrophic obstructive cardiomyopathy (33), or in ST-segment elevation myocardial infarction (34). It is possible that comorbidities may have an important role in the observed association in these studies.

Finally, TyG index was significantly associated with all-cause mortality in our study. In a previous study, Liu et al. (35) demonstrated the association of TyG index with all-cause and cardiovascular mortality. Conversely, in a study by Kim et al., the association between TyG index and all-cause mortality was observed for men but not for women (36). A significant association was also observed between TyG index and cardiovascular mortality in our study. This confirms and extends the results of a previous cross-sectional study where association between TyG index and all-cause and cardiovascular mortality was observed (35). The possible mechanism behind this association remains unclear. It can be speculated that TyG index is closely associated with adverse health risk factors such as obesity, hypertension, and diabetes, which can be contributing factors. It might be that TyG index reflects unhealthy risk factors in general that are strongly linked to mortality risk.

There are several strengths of our study. We used two large cohort studies with prospective design and a long follow-up time linked to national registers of high quality. We were able to explore the longitudinal association between TyG index and arterial stiffness using c-f PWV. Incidence of diabetes and CVD were retrieved through linkages to registers that have been validated (37). A few limitations of the study also need to be considered. Residual confounding cannot be ruled out as this is an observational study. However, information was available for important covariates, which allowed adjustments for parameters. Potentially, another limitation is that covariates were measured once at baseline and might have changed over time. However, in that case it would be more likely to bias our study toward the null.

## Conclusion

In conclusion, the findings of this observational study indicate TyG index to be associated with increased risk of incidence of diabetes, CE, stroke, HF, all-cause mortality and cardiovascular mortality, and with elevated arterial stiffness. As TyG index is a measure of IR, our results indicate that IR is implicated in the risk of future cardiometabolic disorders. TyG index can be easily applied as a useful marker for cardiometabolic risk stratification in the general population. As the TyG index is derived from routine clinical lab investigations, our findings highlight the clinical implications of using TyG index as a marker for IR to routinely screen those at high risk for several adverse health outcomes.

## Data availability statement

The data sets presented in this article are not publicly available because they were used under license for the current study. The data sets are however available from the authors upon reasonable request and with permission of Lund University.

## Ethics statement

The studies involving human participants were reviewed and approved by the Regional Ethics Review Board in Lund (LU 51-90, LU 532-2006, LU 85-2004, and LU 2011-412) and the participants provided written informed consent in MDCS-CV and verbal informed consent in MPP. The study was carried out in accordance with the Helsinki Declaration.

## Author contributions

IFM and SZ designed the study and contributed to the final analysis. IFM performed the statistical analysis and drafted the manuscript. IFM, XB, PN, and SZ contributed to interpretation of the data and critical revision of the manuscript, and reviewed and edited the manuscript. SZ was the guarantor of this work and, as such, had access to all study data and took responsibility for the data integrity and accuracy of the data analysis. All authors approved the final version of the manuscript.
