# Supplementary material for: Triglyceride-glucose (TyG) index is a predictor of arterial stiffness, incidence of diabetes, cardiovascular disease, and all-cause and cardiovascular mortality: A longitudinal two-cohort analysis
Source: Front Cardiovasc Med. 2023 Jan 4;9:1035105. doi: 10.3389/fcvm.2022.1035105 (PMC9846351; doi:10.3389/fcvm.2022.1035105)
Supplement: Supplementary file 1 [file Data_Sheet_1.pdf]

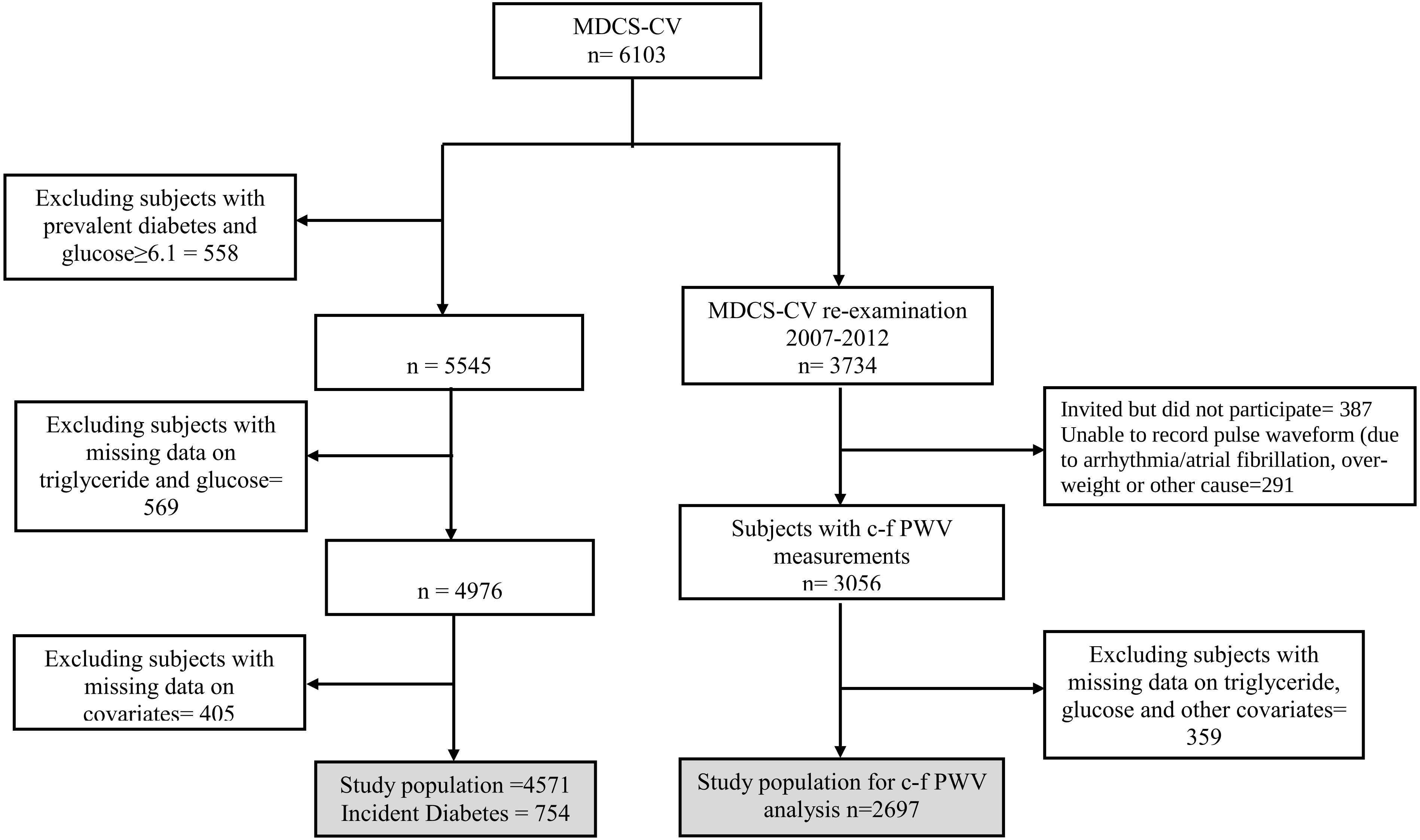

Supplementary Figure 1: Flowchart for the study population in MDCS-CV

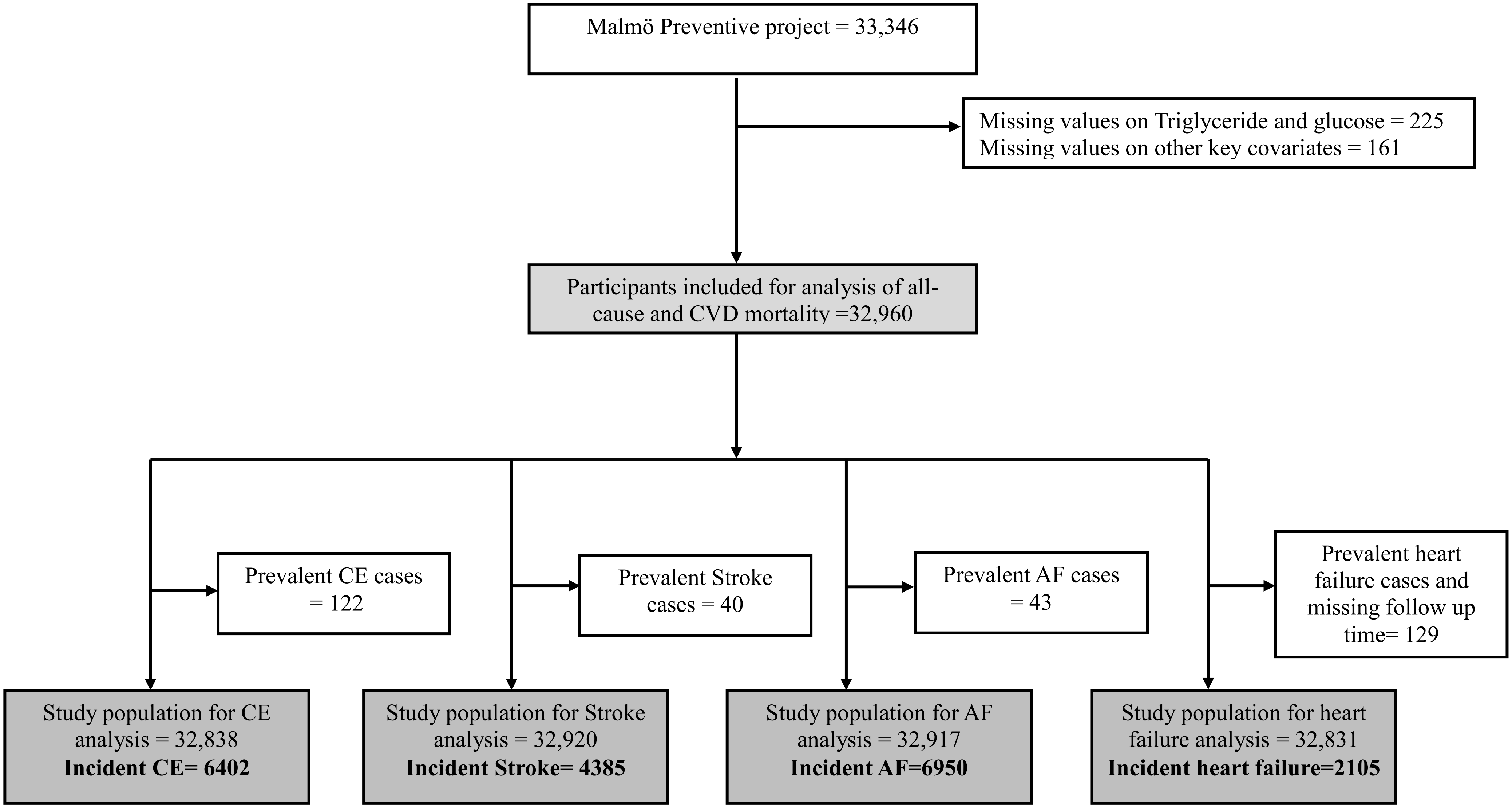

Supplementary Figure 2: Flowchart for the study population in MPP

Supplementary Table 1: Baseline characteristics of participants in the MDCS-CV

	c-f PWV analysis	Incident Diabetes analysis
Number of subjects	2697	4571
TyG index	3.92-5.50	4.55(±0.23)
Age , years	56.05(±5.66)	57.45(±5.96)
Men, n(%)	1028(38.1)	1806(39.5)
Waist , cm	81.70(±11.74)	82.64(±12.25)
Current Smokers n (%)	477(17.7)	1019(22.3)
SBP (mmHg)	137.90(±17.72)	140.53(±18.73)
LDL-cholesterol (mmol/L)	4.13(±0.97)	4.17(±0.98)
HDL-cholesterol (mmol/L)	1.41(±0.37)	1.40(±0.37)
Antihypertensive medication, n(%)	326(12.1)	665(14.5)
Lipid-lowering medication, n(%)	52(1.9)	96(2.1)
CRP, mg/L*	1.20(0.60-2.35)	1.30(0.60-2.70)
HOMA2-IR *	0.80(0.50-1.10)†	0.80(0.50-1.20) ‡
Prevalent diabetes, n(%)	144(5.3)	-
C-f PWV, m/s	10.50(±2.47)	-
Age at follow up, years	72.15(±5.57)	-
Heart rate at follow up, beats/min	62.80(±9.89)	-
MAP, mmHg	95.66(±10.65)	-

Data presented as mean (±standard deviation) unless otherwise stated

*Median (25th-75th percentile)

† n=2673

‡ n=4522

Supplementary Table 2: Baseline characteristics of participants in MPP across the quartiles of TyG index

	Q1	Q2	Q3	Q4	**P for trend**
Number of subjects	8236	8248	8250	8226	
TyG index	3.38-4.38	4.38-4.55	4.55-4.74	4.74-6.70	
Age, years	46.41(±7.73)	45.69(±7.48)	45.24(±7.44)	45.26(±6.92)	<0.001
Men, n (%)	4124(50.1)	5362(65)	5999(72.7)	6756(82.1)	<0.001
BMI (kg/m^2)	23.18(±2.96)	23.96(±3.28)	24.76(±3.52)	26.35(±3.83)	<0.001
Systolic blood pressure (mmHg)	122.37(±14.37)	124.75(±14.85)	126.75(±15.20)	131.21(±16.25)	<0.001
Smokers (current and former), n(%)	4401(43.1)	5123(62.1)	5440(65.9)	5800(70.5)	<0.001
Cholesterol (mmol/L)	5.28(±0.96)	5.54(±0.99)	5.74(±1.05)	6.13(±1.13)	<0.001
Prevalent diabetes, n (%)	28(0.3)	46(0.6)	93(1.1)	640(7.8)	<0.001
Antihypertensive medication, n (%)	276(3.4)	345(4.2)	415(5.0)	759(9.2)	<0.001
High alcohol consumption, n (%)	719(8.7)	910811.0)	1037(12.6)	1386(16.8)	<0.001
Physically activity, men n (%)	5966(72.4)	5077(61.6)	4610(55.9)	4029(49.0)	<0.001
Physically active, women n (%)	2922(35.5)	1988(24.1)	1595(19.3)	1308(15.9)	<0.001

Supplementary Table 3: Association between TyG index and c-f PWV (n=2697)

		95% Confidence interval		
**TyG index**	**Beta coefficient**	**Lower bound**	**Upper bound**	**P value**
**Model 1**	1.50	1.17	1.82	<0.001
**Model 2**	0.77	0.41	1.14	<0.001
**Model 3**	0.60	0.19	1.01	0.005
**Model 4**	0.61	020	1.03	0.018

Model 1: Adjusted for sex, MAP, heart rate and age at follow-up and age at baseline
Model 2: Adjusted for sex, MAP, heart rate and age at follow-up, baseline age, smoking habits, systolic blood pressure, waist circumference, diabetes, use of anti-hypertensive medication and use of lipid-lowering medication
Model 3: Adjusted for sex, MAP, heart rate and age at follow-up, baseline age, smoking habits, systolic blood pressure, waist circumference, diabetes, use of anti-hypertensive medication, use of lipid-lowering medication, HDL and LDL
Model 4: Model 3 + CRP
